# Inoculum Source Determines Acetate and Lactate Production during Anaerobic Digestion of Sewage Sludge and Food Waste

**DOI:** 10.3390/bioengineering7010003

**Published:** 2019-12-23

**Authors:** Jan Moestedt, Maria Westerholm, Simon Isaksson, Anna Schnürer

**Affiliations:** 1 Department of Thematic Studies–Environmental Change, Linköping University, SE 581 83 Linköping, Sweden; jan.moestedt@tekniskaverken.se; 2 Department R&D, Tekniska verken i Linköping AB, SE 581 15 Linköping, Sweden; 3 Department of Molecular Sciences, Swedish University of Agricultural Sciences, BioCenter, SE 750 07 Uppsala, Sweden; maria.westerholm@slu.se (M.W.); simon.isaksson@slu.se (S.I.)

**Keywords:** acetate, lactate, inoculum, food waste, sewage sludge, lactic acid bacteria

## Abstract

Acetate production from food waste or sewage sludge was evaluated in four semi-continuous anaerobic digestion processes. To examine the importance of inoculum and substrate for acid production, two different inoculum sources (a wastewater treatment plant (WWTP) and a co-digestion plant treating food and industry waste) and two common substrates (sewage sludge and food waste) were used in process operations. The processes were evaluated with regard to the efficiency of hydrolysis, acidogenesis, acetogenesis, and methanogenesis and the microbial community structure was determined. Feeding sewage sludge led to mixed acid fermentation and low total acid yield, whereas feeding food waste resulted in the production of high acetate and lactate yields. Inoculum from WWTP with sewage sludge substrate resulted in maintained methane production, despite a low hydraulic retention time. For food waste, the process using inoculum from WWTP produced high levels of lactate (30 g/L) and acetate (10 g/L), while the process initiated with inoculum from the co-digestion plant had higher acetate (25 g/L) and lower lactate (15 g/L) levels. The microbial communities developed during acid production consisted of the major genera *Lactobacillus* (92–100%) with food waste substrate, and *Roseburia* (44–45%) and *Fastidiosipila* (16–36%) with sewage sludge substrate. Use of the outgoing material (hydrolysates) in a biogas production system resulted in a non-significant increase in bio-methane production (+5–20%) compared with direct biogas production from food waste and sewage sludge.

## 1. Introduction

Anaerobic digestion (AD) can be applied for industrial purposes to produce bio-methane (CH_4_), which is used as renewable energy in transportation or for heat and power production. AD can play a key role in reducing fossil fuel use in transportation and industry, while at the same time handling organic waste and producing renewable fertilizer [1]. AD is a complex process that requires several different microbial steps and entails the formation of various intermediary components. The process typically begins with hydrolysis, followed by acidogenesis of complex organic macromolecules into, e.g., volatile fatty acids (VFA), CO_2_, and H_2_ [2]. VFA can be further converted into acetate, CO_2_, and H_2_ in a reaction step called acetogenesis. As a final step in AD, these components are converted into bio-methane by methanogens. The hydrolysis, acidogenesis, and acetogenesis steps are performed by fast-growing bacteria that, in many cases, also thrive at low pH, while methanogenesis is performed by slower-growing bacteria that thrive best at neutral pH. These differences in reaction speed and pH optimum allow AD to be divided into two separate production steps, where H_2_ and VFA are generated in a primary acid reactor and bio-methane in a secondary reactor. This two-stage approach has been shown to optimize the overall AD process and increase methane yield [3,4,5,6,7,8]. It also allows additional applications, such as the production of bio-hydrogen or of a range of VFA that can be extracted and used as “green” chemical feedstock for further conversion [8,9]. The typical VFA composition in an acidic AD reactor is a mixture dominated by acetate, propionate, butyrate, and valerate [10,11,12,13], but it can also be dominated by acetate, butyrate, and H_2_ [14], or mainly consist of lactate and/or acetate [15,16]. The amount and type of acid produced depend on both chemical, technical, and microbiological parameters, often interlinked. Parameters shown to be of importance include, for example, reactor configuration, substrate composition and pre-treatment, hydraulic retention time (HRT), temperature, pH, as well as the choice of inoculum and final microbial composition [10,11,17,18,19,20,21,22].

Among the different acids that can be produced in a two-stage set-up, acetate is a highly interesting compound. In addition to being an energy carrier between different stages within AD, it can be used for the production of value-added products such as biopolymers, commodity chemicals, or fuels, or as a carbon source for denitrification steps at wastewater treatment plants (WWTP) and growing single-cell cultures [23,24]. Today, acetate is predominantly produced (12.9 million metric tons/year) using petrochemical feedstocks through various chemical processes [24]. Production of acetate via microbiological processes during AD can proceed via different metabolic routes, involving different types of microorganism. Various carbohydrates and proteins can be used by fermentative bacteria, resulting in acetate formation but also other longer acids and alcohols, CO_2_, and H_2_ [25]. Monomeric sugars can also be converted by lactic acid bacteria (LAB) to acetate and lactate, in different proportions [15,26]. Another interesting group for acetate production is bacteria performing acetogenesis. These bacteria can use a variety of different organic substrates and inorganic gases (CO, CO_2_, H_2_) and have in common that they use the Wood–Ljungdahl pathway while producing acetate as the main end-product [27]. Depending on growing conditions, some acetogens can also redirect their metabolism toward more reduced end-products such as organic acids and different alcohols [27]. Acetogens can also oxidize VFA (C > 2) to acetate and hydrogen, a process requiring low H_2_ partial pressure to become thermodynamically favorable [28]. In a single-stage process, a low level of hydrogen is ensured by the activity of hydrogenotrophic methanogens. In a hydrolysis reactor with no or low methanogenic activity, hydrogen could potentially be both produced and consumed by different groups of acetogens. 

Knowledge on acid production per se is quite good as it has been studied in many different processes for both single compounds and complex materials and with both single and mixed cultures of microorganisms [8,17,25]. However, less is known about how to direct the process towards the production of a specific VFA from a complex substrate matrix, such as food waste or sewage sludge, owing to the complexity of AD and the wide number of different possible pathways. Moreover, most previous studies have been performed during batch cultivation and only a few studies have evaluated VFA production in continuous mode [20,29]. There is, therefore, a need to identify management strategies for achieving high levels of a specific acid, such as acetate. 

This study investigated the importance of inoculum source (different microbial composition) and substrate composition for the production of VFA, with the focus on acetate. Four semi-continuously fed, laboratory-scale processes were inoculated with sludge from a co-digestion biogas plant or an AD process treating primary and secondary sludge (mixed sludge). Two different materials (food waste and mixed sludge) were used as substrates in a set-up resulting in a distinct combination of inoculum source and substrate. The effects of inoculum origin (expected to have significantly different initial community structure) and substrate on acetate production were then evaluated. VFA production and microbial community structure were followed over time throughout an operating period of 65 days, with the most promising process continued to be operated for an additional 160 days. Moreover, bio-methane potential (BMP) of the resulting hydrolysate was determined and compared with that achieved when using food waste and mixed sludge directly as a substrate. The results obtained were used to estimate the potential for VFA and/or methane production in full-scale processes.

## 2. Materials and Methods

### 2.1. Source of Inoculum

Of the four laboratory continuously stirred tank reactors (CSTR) used in the study, two (CO-F and CO-S) were inoculated with material from a co-digestion plant, and the other two (WW-F and WW-S) were inoculated with material from an AD process fed mixed sludge. The laboratory reactors had an active volume of 6 L. Inoculum for the CO reactors was collected from a co-digestion plant in Linköping, Sweden, that operates a CSTR at 42 °C, an average organic loading rate (OLR) of about 4 kg VS/m^3^/d, and HRT of about 35 days. The co-digestion plant receives food waste from households (50% of incoming wet weight), organic industrial residues (25%), and slaughterhouse waste (25%). Inoculum for the WW reactors was collected from a WWTP anaerobically degrading a mixture of primary and secondary sludge produced during the treatment of wastewater from the city of Linköping (around 150,000 person-equivalents). Its CSTR is operated at an average OLR of 2 kg VS/m^3^/d and HRT of about 20 days at 38 °C.

### 2.2. Experimental Set-Up

All laboratory-scale reactors were fed semi-continuously (once a day, seven days per week). The CO-F and WW-F reactors were fed food waste (total solids (TS) 14.7%, volatile solids (VS) 92%, dissolved organic carbon (DOC) 24,900 mg/L, and total organic carbon (TOC) 39,000 mg/L), which was retrieved at the co-digestion plant. The food waste (annual treatment of approx. 55,000 tons) is macerated into a slurry with particle size <12 mm, diluted with water or waste liquor from food industries, such as washing liquor from a large diary industry, and treated with a Bio-Sep® technique to remove impurities such as metals and plastics. The food waste slurry was collected from a sampling point of the circulation circuit at a heated storage tank (65 °C, HRT 3 days). For the first 20 days after start-up, the reactors were operated at an OLR of 27 kg VS/m^3^/d and HRT of 5 days (week average) in order to rapidly obtain an acidic process (Table 1). These parameters were changed to HRT 10 days (week average) and OLR 13.5 kg VS/m^3^/d, based on results from earlier experiments [6]. Reactors CO-S and WW-S received mixed sludge (TS 8.8%, VS 81%, DOC 5500 mg/L and 21,000 mg/L). The sewage sludge was retrieved from a dewatering band (dewatering mixed sludge, e.g., a mixture of primary- and secondary sludge with TS approx. 2% to 8%). The reactors were operated at OLR 14.2 kg VS/m^3^/d and HRT 5 days (week average) during the initial 50 days. HRT was then decreased to 3 days (week average) and OLR was increased to 23.7 kg VS/m^3^/d, in order to maintain acidic conditions (Table 1). In each set of experiments, one single batch of substrate (kept frozen until use) was used during the entire experiment except in the first three HRTs, when fresh material from the same batch was fed to the reactors. This was done in order to allow for initial inoculation with potential active microorganisms present in the substrate. All reactors were operated for 65 days except for CO-F, which was operated for an additional 160 days, giving in total 225 days of operation. Between days 200 and 225, pure glucose was added to the substrate mixture to observe the effect of adding easily available feed to VFA production. Addition of glucose to CO-F increased the OLR to 16.1 kg VS/m^3^/d, due to an additional ingoing DOC content of 10,700 mg/kg.

### 2.3. Analytical Methods

Reactor volume adjustment and sampling were performed five days per week, prior to daily feeding. Volumetric gas production was measured online with a Ritter milligas counter (MGC-10, Ritter, Waldenbuch, Germany), and methane concentration was determined with a gas sensor (BlueSens, Herten, Germany). Gas was normalized for standard temperature and pressure (1.01325 bar and temperature 273.2 K). Gas composition (CH_4_, CO_2_, H_2_S, O_2_) was further analyzed using a Biogas 5000 device (Geotech Instruments, Coventry, UK). The content of H_2_ in the gas was analyzed with a Micro IV sensor (GfG GmbH, Berlin, Germany). VFA content was analyzed with a Clarus 550 gas chromatograph (Perkin Elmer, Waltham, MA, USA) with a packed Elite-FFAP column (Perkin Elmer, USA) for acidic compounds [30]. Lactic acid was analyzed with HPLC, using a method described elsewhere [31]. Total ammonium nitrogen (NH_4_^+^-N) was analyzed as the sum of NH_4_^+^-N (aq) + ammonia-N (NH_3_-N) (aq) by distillation (Kjeltec 8200, FOSS in Scandinavia, Sweden) in an acidic solution (H_3_BO_3_) and NH_4_^+^-N was then determined by titration with HCl (Titro 809, Metrohm, Herisau, Switzerland) according to the Tecator method for Kjeltec ISO 5664. Kjeldahl-nitrogen was determined using the same procedure and equipment as NH_4_^+^-N, with the exception that the samples were pre-treated with H_2_SO_4_ and subsequently heated to 410 °C for 1 h. The pH was measured with a potentiometric pH meter at 25 °C using a Hamilton electrode (WTW Inolab, Houston, TX, USA). TOC was analyzed according to method SS-EN 1484. The samples were homogenized and acidified with 2 M HCL and 10% H_3_PO_4_ to drive off inorganic carbon, and TOC was determined with Analytik Jena N/C 3100 during combustion of organic carbon and detected as CO_2_ with an NDIR detector. DOC was determined by filtration (0.45 µm) prior to the TOC method. 

Methane potential was determined using an automatic methane potential test system, AMPTS II (Bioprocess Control, Lund, Sweden). The inoculum was collected from the WWTP at Uppsala, Sweden, and degassed for 4 days prior to the test. Each bottle was loaded with inoculum and substrate in a 3:1 ratio (VS basis). To reach an OLR of 3 g VS/L and a working volume of 400 mL (total volume 600 mL), distilled water was added to the mixture. All substrates were operated in parallel triplicates together with three positive controls (crystalline cellulose) and three negative controls (no substrate added). The reactors were then incubated at mesophilic temperature (37 °C) for 15 days while being stirred in cycles of 1 minute, followed by 1 minute of rest.

All confidence intervals presented are calculated with Student’s *t*-test (α = 0.05).

### 2.4. Calculation of Efficiency of Hydrolysis, Acidogenesis, Acetogenesis, and Methanogenesis

The degree of hydrolysis (Equation (1)) and the degree of acidogenesis (Equation (2)) were calculated according to [3], but also taking gas production into consideration according to [32], with the addition of also including CO_2_ production, and based on DOC analyses for liquid phase:

(1) $$Hydrolysis=\frac{DOC_{res}+TC_{Gases}-DOC_{in}}{TOC_{tot}-DOC_{in}}\times 100\;\left(\%\right)$$

(2) $$Acidogenesis=\frac{TOC_{VFA}+TC_{Gases}}{DOC_{res}+TC_{Gases}}\times 100\;\left(\%\right)$$

where

*DOC_res_* = residual dissolved organic carbon,
*DOC_in_* = ingoing dissolved organic carbon,
*TC_Gases_* = total carbon in produced gases,
*TOC_VFA_* = total organic carbon in volatile fatty acids,
*TOC_tot_* = total ingoing organic carbon.

The degree of acetogenesis was calculated as the fraction of TOC_acetate_ in total *TOC_VFA_*. Raw protein was calculated as (Kjel-N − NH_4_^+^-N) × 6.25 and protein hydrolysis as a fraction of raw protein in hydrolysate as compared to substrates [33].

### 2.5. Additional Experiments to Determine Inhibitory Effects of VFA

Possible inhibitory effects of high acetate levels were evaluated by diluting reactor material from CO-F taken at day 200 with tapwater, in a tapwater:hydrolysate ratio of 0:100, 10:90, 25:75, 50:50, and 90:10. A 500 mL subsample of each final dilution of hydrolysate was inoculated in an anaerobic environment using reactors from AMPTSII (Bioprocess Control) with stirring at 38 °C for 48 hours in batch mode. Thereafter, the VFA concentration (specifically acetate) was analyzed. The undissociated (toxic) concentration of acetate was calculated according to Oswald´s law of dilution [34].

### 2.6. Sample Collection, Molecular Analyses, and Sequence Data Processing

Reactor sludge samples for molecular analyses were collected from each reactor on four occasions (days 2, 19, 33, and 57). Four additional samples were taken from reactor CO-F during the prolonged experimental period (i.e., days 135, 156, 177, 226). All samples were stored at −20 °C until further use. DNA extraction, construction of 16S amplicon libraries, and Illumina MiSeq sequencing were carried out on triplicate samples from each sampling point and reactor, as described previously [35]. Sequence data processing was performed as described by Westerholm [36]. In short, contaminating sequences were removed by Cutadapt [37] version 1.13, and sequences were further processed with the software package Divisive Amplicon Denoising Algorithm 2 (DADA2) [38], version 1.4, running in an HPC environment in R, version 3.4.0. Sequences were processed according to the DADA2 pipeline tutorial v. 1.4 with modification according to Appendix B. The forward and reverse reads were truncated at positions 250 and 200 bp, respectively. A maximum expected error of 2 was used to remove low-quality reads, and trimming and filtering were performed jointly on paired reads. Assignment of taxonomy was performed with the DADA2 taxonomy classification, using the Silva training set v128 to classify ribosomal sequence variants (RSVs). The phyloseq package [39] was used to organize the data into a single data object and for the production of graphics in R Studio version software (http://www.r-project.org, TeamR RStudio, 2016) as described previously [36]. In addition, a plot of richness estimates was created using the *plot_richness* function and a heatmap was created using the plot_heatmap function in the phyloseq package. Principal coordinate analysis (PCoA) plots of microbial community profiles were generated using Bray–Curtis weighted UniFrac distance measures. Constrained ordination was performed to evaluate associations between reactor parameters and changes in community composition. Permutational ANOVA (PERMANOVA) was performed to evaluate the effect of operating parameters on microbial community structure using the adonis functions in the vegan package [40] and significant (*p* < 0.05) parameters were included in canonical correspondence analysis (CCA) plotting. The ordination axes were constrained to linear combinations of reactor variables and plotted as arrows onto the ordination. The statistical significance of differences between reactors and over time was determined using ANOVA. Phylogenetic assignment at the genus level was evaluated using the Basic Local Alignment Search Tool (BLAST) algorithm [41] provided by the National Center for Biotechnology Information (NCBI; http://www.ncbi.nlm.nih.gov). 

Raw sequences were submitted to the NCBI Sequence Read Archive (SRA) under the study accession number PRJNA575652.

## 3. Results and Discussion

**Figure 1:**
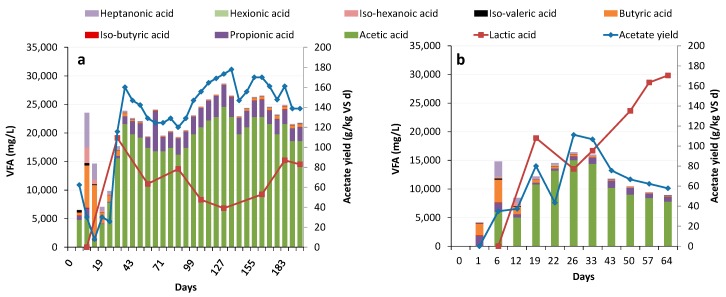
Volatile fatty acid (VFA) composition and acetate yield in reactors (**a**) CO-F (reactor inoculated from co-digestion plant fed with food waste) and (**b**) WW-F (reactor inoculated from wastewater treatment plant fed with food waste).

**Figure 2:**
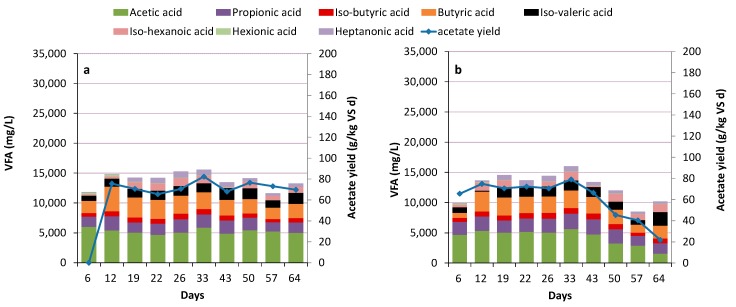
Volatile fatty acid (VFA) composition and acetate yield in reactors (**a**) CO-S (reactor inoculated from co-digestion plant fed with sewage sludge) and (**b**) WW-S (reactor inoculated from wastewater treatment plant fed with sewage sludge).

The food waste-degrading reactors (CO-F and WW-F) had very low methane content (<1%) during the initial nine days when operating at 27 kg VS/m^3^/d and HRT 5 days. pH decreased quickly in both reactors with some differences depending on the initial inoculum, from 7.8 to 5.2 in CO-F and 4.6 in WW-F. In this period, H_2_ was produced with a concentration peaking at 20% and 15% of gas produced in CO-F and WW-F, respectively. After day 20, the H_2_ level stabilized and represented around 1% of the gas produced. The high H_2_ level coincided with a VFA profile dominated by acetate and butyrate (Figure 1), which is typical for dark fermentation [26]. Propionate and heptonate were also formed in high concentrations (Figure 1). However, from around day 20, in line with the drop in H_2_ level, acetate and lactate became the dominant acids in both reactors, but with differences in the total amounts depending on the initial inoculum. At this time, the pH reached 3.8 ± 0.1 in WW-F and 4.0 ± 0.2 in CO-F and total gas production was very low (<1 g C_in gas_/L_substrate_ d in WW-F; <2 g C_in gas_/L_substrate_ d in CO-F), in line with previous studies on hydrolysis of food waste [6,10,20,42,43]. In contrast, in the sludge reactors (CO-S and WW-S), methane content and pH did not drop to similarly low levels when operating at initial parameters of OLR 14.2 kg VS/m^3^/d and HRT 5 days. The methane content of the gas produced declined after 2 HRT (10 days), from 60% to stabilize at 15–20% in both reactors, while pH remained at 6.1 ± 0.2 throughout the main part of the experiment and the gas produced contained <2% H_2_. However, from day 50 until 65 pH increased in WW-S (6.7 ± 0.7) while it remained stable in CO-S. The increase in pH occurred simultaneously as VFA-concentration decreased, and methane production started to increase. The VFA profile for both sludge reactors was different from that of the food waste reactors and included acetate, propionate, butyrate, and valerate, but no lactate (Figure 2). In contrast to the food waste processes, this profile also remained stable, although the absolute concentration decreased at the end of the experiment in WW-S (Figure 2). 

As further discussed below, one possible explanation for the differences in the observed VFA profiles between the processes operating with the different substrates was the differences in pH, suggested in several studies to be a key factor determinative for the type of organic acid produced [17,20,21].

### 3.1. Hydrolysis Efficiency

The ingoing TOC for mixed sludge was 21,000 mg/L and dissolved carbon in the substrate measured as DOC was 5500 mg/L. These results revealed that about 26% of the carbon in the mixed sludge was in dissolved form when entering the reactors (Table 2). In both mixed sludge processes (WW-S and CO-S), the degree of hydrolysis was around 15%. For WW-S, a decrease in the degree of hydrolysis was seen from day 50; however this was likely due to the consumption of released DOC caused by the increasing methanogenic activity (Table 2). Specifically, hydrolysis of proteins into free ammonium-nitrogen appeared to be efficient and the level reached 53–59% for WW-S and 57–65% for CO-S. In total, the soluble fraction of TOC, including both ingoing DOC from the substrate and additionally released DOC from the acidic stage, was 41% for both reactors on days 0–50. The outgoing total soluble fraction of TOC was similar to the hydrolysis efficiency (42%) found in a previous study during semi-continuous acidification and hydrolysis of mixed sludge [32,44]. In that study, the hydrolysis efficiency increased with increasing pH and maximum acidification, and hydrolysis efficiency was reached at pH 8.9 and 9.9, respectively.

For the processes fed food waste (CO-F and WW-F), the overall hydrolysis efficiency was considerably lower (2% and 7% in WW-F and CO-F respectively) than in the sludge-fed processes (Table 2). Furthermore, only 15–17% of the protein content was hydrolyzed, irrespective of the inoculum source, which was considerably lower than the fraction of proteins hydrolyzed in the sludge reactors (53–65%) (Table 2). This result was unexpected, as the protein mass in food waste ought to be more bioavailable than the material in mixed sludge, which is repeatedly pre-processed in various degradation steps in both the human gastrointestinal tract and the WWTP before entering the AD process. However, in food waste, the majority (64%) of the ingoing TOC (39,000 mg/L) was already in dissolved form (25,000 mg/L). Thus, even though the hydrolysis efficiency was low, the total outgoing DOC from the food waste processes was higher than for the sludge processes. The ingoing DOC of 64% and an additional 2–7% resulted in almost 70% total soluble fraction of TOC in the food waste reactors, indicating very high substrate availability for further treatments. The degree of hydrolysis was still much lower than observed before by Feng et al. [20] and Wu et al. [3], who reported overall hydrolysis efficiency of ca 40% in the AD of food waste. However, in these studies, the ingoing DOC represented less than 50% of total TOC and, therefore, the total outgoing soluble fraction of TOC was similar to that achieved for food waste in the acidic stage in the present study. The reason for low hydrolysis with food waste could be low activity by hydrolytic bacteria. Since almost no ammonium-nitrogen (<200 mg NH_4_^+^-N/L) was present in the hydrolysate, the vast majority of proteins passed through this process without being hydrolyzed into smaller molecules (e.g., NH_4_^+^). Similarly, Yin et al. [4] observed higher hydrolysis from carbohydrates than from proteins in the hydrolysis of food waste.

### 3.2. Acidogenesis and Acetogenesis

**Figure 3:**
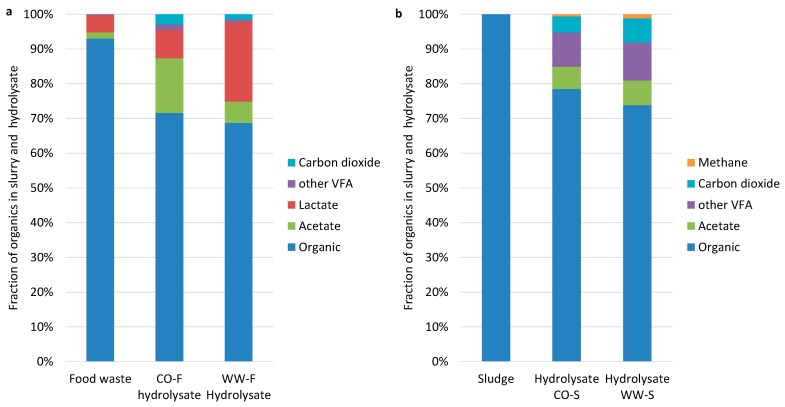
Composition of ingoing substrate and outgoing hydrolysate from reactors fed (**a**) food waste (CO-F, WW-F) and (**b**) sewage sludge (CO-S, WW-S). Mass of outgoing gases is included in the hydrolysate.

The mixed sludge reactors initially had acidogenesis efficiency corresponding to 66–70% (Table 2), with no significant difference between the processes. This indicates that the majority of DOC released from hydrolysis and DOC present in the substrate were converted into acids. However, over time the two processes deviated and for WW-S, the acidogenesis efficiency finally decreased to 48%. In this process, the VFA concentration at day 33 was 16.0 g/L, but it decreased to 10.2 g/L at day 58, of which acetate represented only 1.5 g/L. Simultaneously, methane production increased (Figure 1, Figure 2 and Figure 3; Table 2). In CO-S, the acidogenesis efficiency was more stable and slightly higher than in WW-S (Table 2), with a total acid concentration of around 14.0 ± 0.9 g/L, acetate around 5 g/L and pH 5.9–6.1 throughout the experiment. The acetate fraction in CO-S represented 37% of total VFA and the yield corresponded to 70 g/kg VS. In WW-S, an equivalent level of acetate was reached during the first half of the experiment, but thereafter it declined to 22 g/kg VS, representing 15% of total VFA, by the end of the experiment. In the sludge reactors, the HRT was lowered after 50 days of operation in an attempt to wash out methanogens in WW-S. However, methanogenesis prevailed and reached 7% of ingoing TOC in WW-S, even at HRT of 3 days. Moreover, the pH increased from 6.1 to 7.0 by the end of the experiment, indicating that acidification of mixed sludge without pH regulation is difficult to obtain, particularly with inoculum from WWTP. High production of acetate was hence difficult to obtain with mixed sludge, and mixed-acid fermentation instead dominated. These results somewhat contradict previous findings of acetate levels up to 50–60% of total VFA in AD with mixed sludge [45,46,47]. However, those studies reached much lower total VFA concentrations (3–8 g/L) [45,46,47], especially compared with CO-S (14.0 ± 0.9 g/L), and hence the actual acetate concentration was similar or even higher in the present study. The observed difference between the processes, using the same substrate and operating conditions, suggests that the inoculum source played a major role in the efficiency of acidogenesis of the sludge. Differences in inoculum could thus also be one explanation for observed differences in process performance between studies.

The reactors fed food waste had somewhat different acidogenesis efficiency in the initial phase of operation, with a lower value for the process initiated with the WWTP inoculum. However, from day 20 onwards, the processes became similar and reached 56–65% from day 20 onward (Table 2). The acid yields were higher than the sewage sludge reactors since food waste had a higher DOC concentration than the sludge (Figure 1 and Figure 2). Several studies evaluating the effect of pH on acidogenesis and acetogenesis from food waste have found correlations between low pH and high hydrolysis of carbohydrates, while high pH increases hydrolysis of proteins, and thus release of ammonia [10,11,42]. Similarly, reactors CO-F and WW-F, with pH around 4, showed high acidogenesis of carbohydrates, while the release of NH_4_^+^-N was low. The importance of the inoculum source for acetate production was illustrated by a considerably higher yield in CO-F than WW-F reaching 151 and 75 g/kg VS, respectively. The acetate concentration was very high in both reactors (86–89% of total VFA, excluding lactic acid) from day 20. This supports the suggestion that low pH (in this case, uncontrolled) could be a key factor for reaching a high fraction of acetate. Differences in pH could also have caused the difference in acetogenesis efficiency between the sludge and food waste reactors, with the latter having both a lower fraction of acetate and higher pH (pH > 5.9). The high acetate levels and low pH in the food waste reactors could also have been the result of the high concentration of lactic acid in those reactors. Since lactate has low pKa, the pH will naturally decrease below 4, which could benefit acetate production. In WW-F with a pH of 3.8, the lactate concentration increased throughout the experimental period, to reach 30 g/L (Figure 3), which corresponded to 76% of DOC in acidogenesis. Lactate was lower in CO-F with pH 4.0 (10 g/L) and corresponded to 29% of DOC in acidogenesis (Figure 3).

#### Limiting Factors—Undissociated Acetate and Ingoing DOC

**Figure 4:**
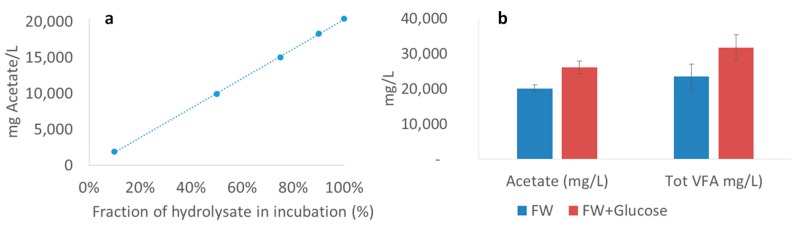
(**a**) Correlation between fraction of hydrolysate after two days of inoculation and absolute acetate concentration (R^2^ = 0.9997). (**b**) Concentration of acetate and total volatile fatty acids (VFA) with only food waste (FW) (blue) compared with FW plus extra dissolved organic carbon (red).

Reactor CO-F had the highest acetate yield and was kept in operation for 200 days (20 HRTs) in order to evaluate the long-term stability of the process. The process was quite stable, converting around 15% of the ingoing TOC to acetate and with an outgoing acetate concentration varying between 17 and 28 g/L. A possible limitation for increased acetate yields could be inhibitory effects of undissociated VFA. The average concentration of 20 g acetate/L is equivalent to 18.6 g undissociated acetate/L at pH 3.9, which is far above the value in other studies showing inhibitory effects [48,49]; e.g., Xiao et al. [48] observed that about 2 g/L at pH 5.5 could reduce acetate production by 60% compared with when a smaller fraction of undissociated acids was present. To obtain information about possible toxicity effects, a batch dilution experiment was performed. The results showed a linear correlation (R^2^ = 0.9997) between acetate concentration after two days of incubation and the fraction of hydrolysate (Figure 4a). Hence dilution of acetate (i.e., reducing the undissociated, toxic concentration) did not result in higher acetate production, which theoretically would have been the case in case the acetate level was limited by a toxic effect (Figure 4a). This shows that the concentration of undissociated acids was not detrimental to reaching higher acetate concentration in the processes in this study. 

Another limiting factor for optimizing acetate production from the food waste could be the level of available DOC in the substrate. One possible way to increase available DOC would be to pretreat the substrate, shown in a previous study to increase the DOC and VFA production from source-separated organic waste [43]. To test the effect of higher DOC on acetate production, additional DOC in the form of glucose was added to the substrate for CO-F during the last 25 days of operation. The ingoing DOC was already 25,000 mg/L, but following the glucose addition, the concentration was increased to 35,700 mg/L, which significantly increased both acetate and residual VFA concentration (Figure 4b). This confirms that it was not the concentration of undissociated acids that was the limiting factor, but rather the amount of easily convertible substrate. Still, while the absolute concentration of acetate increased with additional DOC in the substrate, the actual acetate yield from the added substrate (mg/g VS) was constant. This shows that it is possible to obtain higher acetate concentration with a substrate containing a large fraction of DOC, although in this case, the expected yield per amount of added organic material appeared to be constant (~150 g/kg VS).

### 3.3. Microbial Community Structure

#### 3.3.1. Importance of Inoculum Source

**Figure 5:**
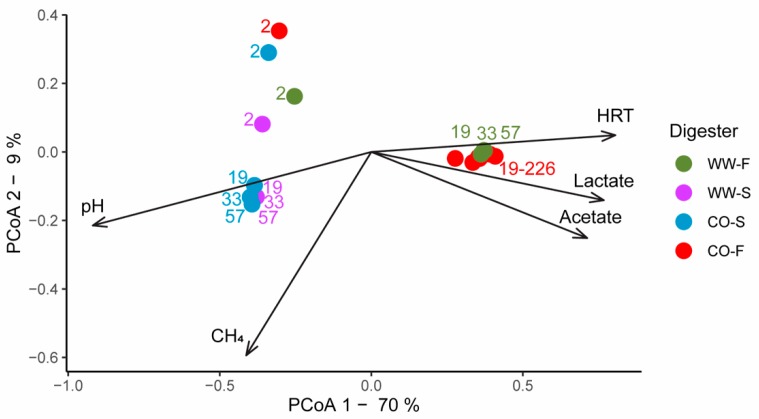
Principal coordinate analysis (PCoA) plot of microbial community structure using weighted UniFrac. Reactor parameters significantly associated with changes in microbial community structure are plotted as vectors, where the length and direction indicate the contribution of the variable to the principal components. Variable HRT stands for hydraulic retention time.

**Figure 6:**
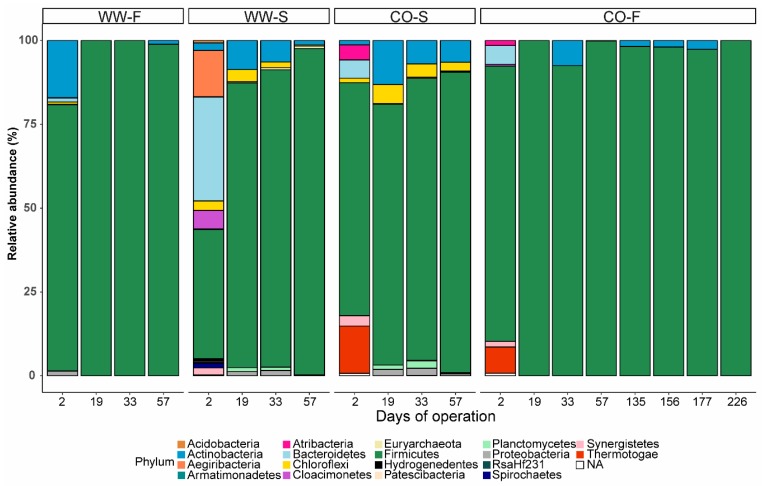
Relative abundance of microbial phyla (based on total bacterial and archaeal sequences) in reactors inoculated with sludge from a co-digestion plant (CO) or an anaerobic digestion process fed mixed sludge (WW) and fed food waste (F) or sewage sludge (S). Days of operation at the point of sampling are given on the *x*-axis.

The microbiology of the biogas plants from where the inocula were taken has been thoroughly analyzed in previous studies [50,51]. These investigations have shown that the high-ammonia co-digestion plant has syntrophic acetate oxidation (SAO) and hydrogenotrophic methanogenesis as the dominant reaction pathway for methane formation, whereas the low-ammonia AD process of mixed sludge is dominated by acetotrophic methanogens. The effect of inoculum source in processing food waste for acid production has also been evaluated in previous studies, although mainly in batch mode. Those studies identified inoculum source as a detrimental factor for process performance [4,18,21,42], as also found in the present study. However, based on the Illumina sequencing results, the inoculum source appeared to have minor effects on the development of the microbial community structure. The analyses demonstrated relatively similar overall microbial community structure at two days of operation, with dominance of the phylum Firmicutes and minor levels of Thermotagae (8–14%), Synergistetes (2–3%), Bacterioidetes (5%), and Atribacteria (1–5%) in the processes with co-digestion plant inoculum (Figure 5 and Figure 6). In the processes inoculated with sludge, Firmicutes (79%) was accompanied by Actinobacteria (17%) in WW-F, whereas Firmicutes (38%), Bacteroidetes (31%), and Aegiribacteria (14%) dominated in WW-S. However, differences in the microbial community caused by the inoculum in CO and WW reactors diminished over the course of the operation and the community structure became more structured based on the substrate (Figure 5), as discussed further in the next section.

Although no strong effect of inoculum source on microbial community structure was observed, there were differences in process performance, such as higher methanogenic activity in WW-S (7% of TOC) compared with CO-S (2% of TOC) (Table 2). This difference indicates that the methanogenic community originating from an inoculum dominated by SAO and hydrogenotrophic methanogenesis (i.e., CO-S) was easier to wash out at high OLR than an acetotrophic community (i.e., WW-S). SAO-driven processes are known to have slower growth rates than acetotrophic methanogenesis [52], a possible explanation for the observed results. Still, as mixed sludge is a rather recalcitrant material where hydrolysis limits acid production [53], it is clearly difficult to load the process to sufficiently high level to reach overload of the methanogenic step, irrespective of methanogenesis pathway. Consequently, even though the acetate to total VFA ratio was similar for the two processes fed sludge, the higher methanogenic activity in WW-S likely explains the lower total VFA yield. For food waste, with a higher degree of DOC in the ingoing TOC, hydrolysis was less important for reaching high VFA production. In these processes, a difference was noted for the processes started with the different inocula, with the inoculum from the co-digestion plant resulting in higher acetate yield. A possible explanation for this is the higher hydrogen consumption rate by acetogenesis, which would facilitate oxidation of longer-chain VFA to acetate [54].

#### 3.3.2. Effect of Substrate on Microbial Community Structure

**Figure 7:**
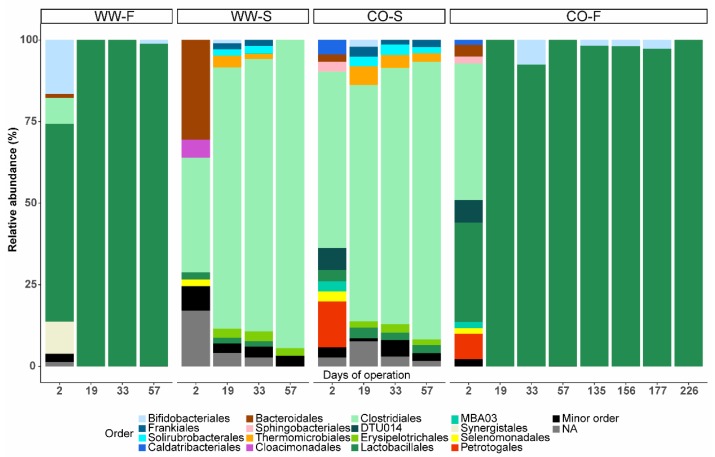
Relative abundance of microbial order (based on total bacterial and archaeal sequences) in reactors inoculated with sludge from the co-digestion plant (CO) or an anaerobic digestion process fed mixed sludge (WW) and fed food waste (F) or sewage sludge (S). Days of operation at the point of sampling are given on the *x*-axis.

Although the microbial communities in all four reactors were similar at the phylum level, with a vast majority belonging to Firmicutes (90–100%; Figure 6), differences depending on substrate became obvious at lower taxonomic rank after 19 days of operation (Figure 5 and Figure 7). The microbial community in the reactor inoculated with sludge and fed food waste (WW–F) were already diverged at day 2 from the other process inoculated with similar sludge (WW-S) and became highly similar to the community in the CO-F reactor (Figure 5). Similarly, from day 19 onwards, the reactors inoculated with material from the co-digestion plant diverged and became highly similar to the process fed the same substrate (Figure 5).

#### 3.3.3. Food Waste Reactors

In both WW-F and CO-F, the genus *Lactobacillus* (order Lactobacillales) clearly dominated (92–100%) and was only accompanied by minor levels of the genus *Aeriscardovia* (order Bifidobacteriales <5% from day 33 (Figure 7 and Supplementary Figure S1). The genus *Lactobacillus* was represented by sequence variants, for which Blast searches revealed a relationship (93–97% gene sequence similarity) to *Lactobacillus amylolyticus*. This species has been isolated from beer malt [55] and is known to efficiently convert carbon (including starchy materials) to lactic acid under anaerobic conditions [56]. Similarly, in an earlier study of acid production from food waste during semi-continuous operation, *Lactobacillus* dominated the processes, with a smaller fraction of Bifidobacteriaceae (*Aeriscardovia* at genus level). In that study, a combination of acetic acid and/or lactic acid was produced [15]. Likewise, Lactobacillus was also highly abundant during continuous VFA production from food waste at low pH (3.2–4.5) in the study by Feng et al. [20]. Lactobacillus has also been also shown to dominate during batch-wise VFA production from food waste, with comparably higher abundance at acidic compared or neutral and alkaline conditions [21,57]. The observed high abundance of *Lactobacillus* is likely due to the character of the food waste, with high levels of soluble organic matter. However, the substrate per se also represents a source of active microorganisms, which is an additional factor potentially influencing the microbial composition and consequently the fermentation products obtained [13,16]. In the study by Yin et al. [13], lactate was produced as a major product from non-treated food waste, while no lactate was formed after thermal treatment (and thus sterilization) of the food waste. Similarly, since the reactors in the present study were fed non-hygienized food waste, the high lactate concentration could be due to the activity of microorganisms originating from the substrate.

*Lactobacillus* spp. can utilize a wide range of sugars as the carbon source, while producing either mainly lactate (homofermentation) or lactate, acetate, and ethanol (heterofermentation), while one species within this genus is able to convert lactate to acetate and formate (and in rare cases form H_2_) [58]. Thus, the formation of acetate from lactate has been observed while fermenting food waste at regulated pH 7, with associated H_2_ production [59]. These three routes may, therefore, have occurred simultaneously to differing extents in the food waste reactors, explaining the divergent patterns of lactic and acetic acid production in the reactors (Figure 2). From day 57 onward, CO-F produced a molar ratio of acetic acid:lactic acid of about 2:1. In WW-F, this ratio was instead about 1:2.5. Neither of these patterns can be explained by the occurrence of simply homo- or heterofermentation, which would theoretically yield only lactate or a 1:1 ratio of lactate to acetate. Thus, some of the lactic acid produced was most probably degraded into acetate to different extents, yielding the observed molar ratios. The lactic acid concentration increased in WW-F after day 33, which correlated with a drop in pH from 3.9 to on average 3.6 between days 33 and 64. In CO-F, the pH remained between 3.9 and 4.2 and here, the acetate level was comparably higher. A similar pattern was seen in the study by Feng et al. [20], where the proportion of lactate to acetate during continuous fermentation of food waste increased with decreasing pH (<4, controlled). In that study, the acetate level increased at pH above 4.5, in line with an increase in the relative abundance of Bifidobacteria. This connection between acetate production and abundance of Bifidobacteria could not be confirmed in the present study, showing similar levels in both CO-F and WW-F. The decrease in pH could have been a consequence of the increased lactic acid production, or of low pH decreasing the capability for lactic acid conversion into acetate. This has been observed for another *Lactobacillus* species (*L. bifermentans*), which requires pH > 4 for activity [58,60]. A similar correlation between acetic acid or lactic acid as the major product and pH has also been observed during acid production from food waste [15].

#### 3.3.4. Sewage Sludge Reactors

The communities in both reactors fed mixed sludge diverged from those in the reactors fed food waste. They were dominated by the order Clostridiales (representing up to 85–94% at day 57), followed by smaller fractions of Lactobacillales, Erysipelotrichales (phylum Firmicutes), Thermomicrobiales (Chloroflexi), Solirubrobacterales, and Frankiales (Actinobacteria) (<3% each) (Figure 7). Investigations at genus rank revealed an increased relative abundance of *Roseburia* (family Lachnospiraceae) and *Fastidiosipila* (family Ruminococcaceae), representing 44–45% and 16–36% of the total community at day 57, respectively (Supplementary Figures S1 and S2). *Roseburia* is able to utilize carbohydrates and acetic acid and produce butyric and lactic acids [61,62,63], and could thus have been the main producer of the high level of butyric acid found in the sludge reactors (Figure 1). *Fastidiosipila* may be involved in production of acetic and butyric acids [64] and has previously been identified as a major genus in mesophilic (35–37 °C) anaerobic digesters treating a variety of wastes, including the organic fraction of municipal solid waste [65], food waste [66,67], landfill leachate [68], and sewage sludge [69].

As indicated at the family level, minor levels (≤7%) of Peptostreptococcaceae, Family XIII, and Eubacteriaceae (all belonging to the order Clostridiales) were detected in reactors WW-S (days 19–33) and WW-F (days 19–57; Figure S2). In WW–S, several of the low-abundance bacteria (Peptostreptococcaceae, Family XIII, Eubacteriaceae) decreased below the detection level at the last sampling point (day 57). Members of the families Peptostreptococcaceae and Eubacteriaceae are known to ferment carbohydrates and proteins to various acids or sugars into acetate, respectively [70,71]. Peptostreptococcaceae are able to produce acetic acid from a variety of sugars and glycerol, as well as from CO_2_ and H_2_. Hence, at the last sampling point, when both carbohydrate- and protein-hydrolyzing families disappeared, hydrolysis, acidogenesis, and acetogenesis decreased in efficiency in the WW-S reactor (Table 2). This shift co-occurred with the increase in pH from 6.0–6.1 to 7.0. Since all Peptostreptococcaceae have optimum pH around 7, it is possible that the increasing pH was a consequence of the microbial shift (and lower acidogenic efficiency), rather than the converse. 

Microbial community richness and evenness were relatively similar in all processes, and no drastic change over time was observed (Supplementary Figure S3).

### 3.4. Methane Potential

**Figure 8:**
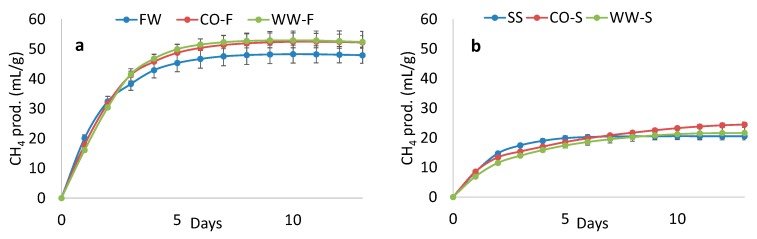
Volumetric bio-methane production from (**a**) food waste (FW) and hydrolysate from reactors CO-F and WW-F, and (**b**) sewage sludge (SS) and hydrolysate from reactors CO-S and WW-S. Error bars indicate the calculated standard deviation for daily values.

In order to evaluate the effect of two-stage AD on the overall methane yield, each hydrolysate and the respective untreated substrate were evaluated using BMP tests. Since CO_2_ and bio-methane were produced during the acidic stage (resulting in loss of mass) and organic material had partly been converted to volatile compounds, the comparison between fresh substrate and hydrolysate was based on volumetric production (mL CH_4_/g material), rather than per g VS. Compensation for mass loss by the gases produced is important to avoid misinterpretation of the BMP result. Bio-methane production from the substrates and hydrolysates was significantly higher for food waste (47–52 mL CH_4_/g material) than for mixed sludge (21–25 mL CH_4_/g material; Figure 8). Differences in methane yield potential between hydrolysate and substrate in the present study were non-significant, due to high variation in the results. However, these results can still assist in interpreting the effect of operating two-stage AD on bio-methane yield. The hydrolysate from all reactors gave 5–20% higher methane yield than the untreated material, indicating that two-stage AD could be very positive for the overall methane yield for both mixed sludge and food waste. Similarly, Luo et al. [7] obtained an 11% increase in overall energy output on applying two-stage operation, but with hydrogen production in the first stage.

For the processes treating mixed sludge, which showed a higher degree of hydrolysis than when food waste was used, different gas production kinetics for treated and untreated material were expected, with faster kinetics from hydrolysate due to release of DOC in the acidic stage. In fact, the untreated mixed sludge actually gave a higher gas production rate during the initial five days (Figure 8). One reason for this could be that persistent methanogenic activity during the acidic stage of operation in WW-S and CO-S consumed most readily available DOC before the BMP test. This assumption is supported by the faster methane production kinetics in CO-S compared with WW-S, with the former having lower bio-methane production in the previous semi-continuous experiment. For the food waste system, the kinetics were similar for all fractions during the first four days, but thereafter, the hydrolysates resulted in higher bio-methane production [15].

### 3.5. Potential Acid Production

Results for reactor CO-F, with the highest acetate yield per kg VS, were used to estimate acetate production potential from food waste in an industrial-scale scenario. In the co-digestion plant (Linköping) from which the substrate and inoculum were collected, about 50,000 tons of food waste are treated annually. On average, about 28% of the ingoing material (weight basis) is organic material, i.e., VS. Reactor CO-F had an average conversion efficiency of 16.2% of the ingoing VS, and hence an expected 2300 tons of acetate could be produced from the food waste treated in the Linköping co-digestion plant by applying a two-stage operation. The market value of this quantity of acetate is substantial (2.7–3.8 million USD) [25]. However, production and extraction of acetate would decrease the biogas production equivalent to 625,000 kg CH_4_. This corresponds to 5,650,000 kWh of energy and a market value of about 1.3 million USD. The production of acetic acid could hence be profitable if it could be extracted in an efficient manner from the rather complex matrix of the hydrolysate, an issue that needs to be further studied.

## 4. Conclusions

This study clearly demonstrates that both substrate and inoculum have major effects on the acidic stage of AD. High VFA and acetate yields were difficult to obtain when treating sewage sludge, indicating that the efficiency of hydrolysis and acidogenesis was too low to achieve acidification of the process, although protein hydrolysis efficiency was higher with sewage sludge than with food waste. When food waste was used as feedstock, acidification and low pH were obtained. At low pH, protein degradation was very low, while available DOC in the form of carbohydrates and fat was apparently readily converted into VFA. The process using inoculum from WWTP produced high levels of lactate (30 g/L) and acetate (10 g/L), while the process using co-digestion plant inoculum had higher acetate (25 g/L) than lactate (15 g/L) levels. Despite the reactors having similar microbial communities, the acids produced differed between the food waste reactors, indicating that the resulting lower pH in WW-F was the cause of the higher lactate production. The microbial communities developed during acid production mainly consisted of the genera *Lactobacillus* (92–100%) when fed food waste, and *Roseburia* (44–45%) and *Fastidiosipila* (16–36%) when fed sewage sludge waste. Use of the hydrolysates in a biogas production system resulted in a non-significant increase in bio-methane production compared with directly using the substrate for biogas production (5–20% increase).

## Figures and Tables

**Table 1:** Origin of inoculum, substrate, organic loading rate (OLR), and hydraulic retention time (HRT) for the different reactors (week averages). Values in brackets refer to after day 20 for reactors CO-F and WW-F, and after day 50 for reactors CO-S and WW-S.

	WW-S	CO-S	WW-F	CO-F
Inoculum source	WWTP	Co-digestion	WWTP	Co-digestion
Substrate	Sewage sludge	Sewage sludge	Food waste	Food waste
OLR (kg VS/m^3^/d)	14.2 (23.7)	14.2 (23.7)	27.0 (13.5)	27.0 (13.5)
HRT (days)	5 (3)	5 (3)	5 (10)	5 (10)

**Table 2:** Efficiency of the different steps in anaerobic digestion for the reactors fed mixed sludge (WW-S and CO-S) and the reactors fed food waste (WW-F and CO-F). TOC = total organic carbon, DOC = dissolved organic carbon, VFA = volatile fatty acids, nd = not determined.

	WW-S		CO-S		WW-F		CO-F		
Time period	0–50	50–65	0–50	50–65	0–20	20–65	0–20	20–200	days
HRT ^a^	5	3	5	3	5	10	5	0	days
Hydrolysis	15%	10%	15%	15%	1%	2%	6%	7%	of TOC in
Acidogenesis	66%	48%	71%	61%	38%	65%	61%	56%	of DOC
Acetogenesis	38%	25%	37%	40%	37%	21%	40%	58%	of VFA
Protein hydrolysis	53%	59%	65%	57%	-	15%	-	17%	of raw protein in
Methanogenesis (1st stage)	4%	7%	2%	2%	1%	0%	1%	0%	of TOC in
Methanogenesis (2nd stage)	nd	59%	nd	60%	nd	44%	nd	46%	of TOC in

^a^ The HRT for WW-S and CO-S was decreased from 5 to 3 days after 50 days, and for WW-F and CO-F, it increased from 5 to 10 days after 20 days.

## Supplementary Materials

The following are available online at https://www.mdpi.com/2306-5354/7/1/3/s1, Figure S1: Heatmap illustrating the relative abundance of genera in reactors inoculated with sludge from co-digestion plant (CO) or from an anaerobic digestion process fed mixed sludge (WW), and fed food waste (F) or sewage sludge (S). Genera discussed in the text are shown in red. Days of operation at point of sampling are given on the x-axis, Figure S2: Relative abundance of microbial families (based on total bacterial and archaeal sequences) in reactors inoculated with sludge from co-digestion plant (CO) or from an anaerobic digestion process fed mixed sludge (WW), and fed food waste (F) or sewage sludge (S). Days of operation at point of sampling are given on the x-axis, Figure S3: Microbial richness and evenness indices in the digesters inoculated with sludge from co-digestion plant (CO) or from an AD process fed mixed sludge (WW) and fed food waste (F) or sewage sludge (S). Day number in the study period is given on the x-axis.
