# Supplementary material for: Inoculum Source Determines Acetate and Lactate Production during Anaerobic Digestion of Sewage Sludge and Food Waste
Source: Bioengineering (Basel). 2019 Dec 23;7(1):3. doi: 10.3390/bioengineering7010003 (PMC7175179; doi:10.3390/bioengineering7010003)
Supplement: Supplementary file 1 [file bioengineering-07-00003-s001.pdf]

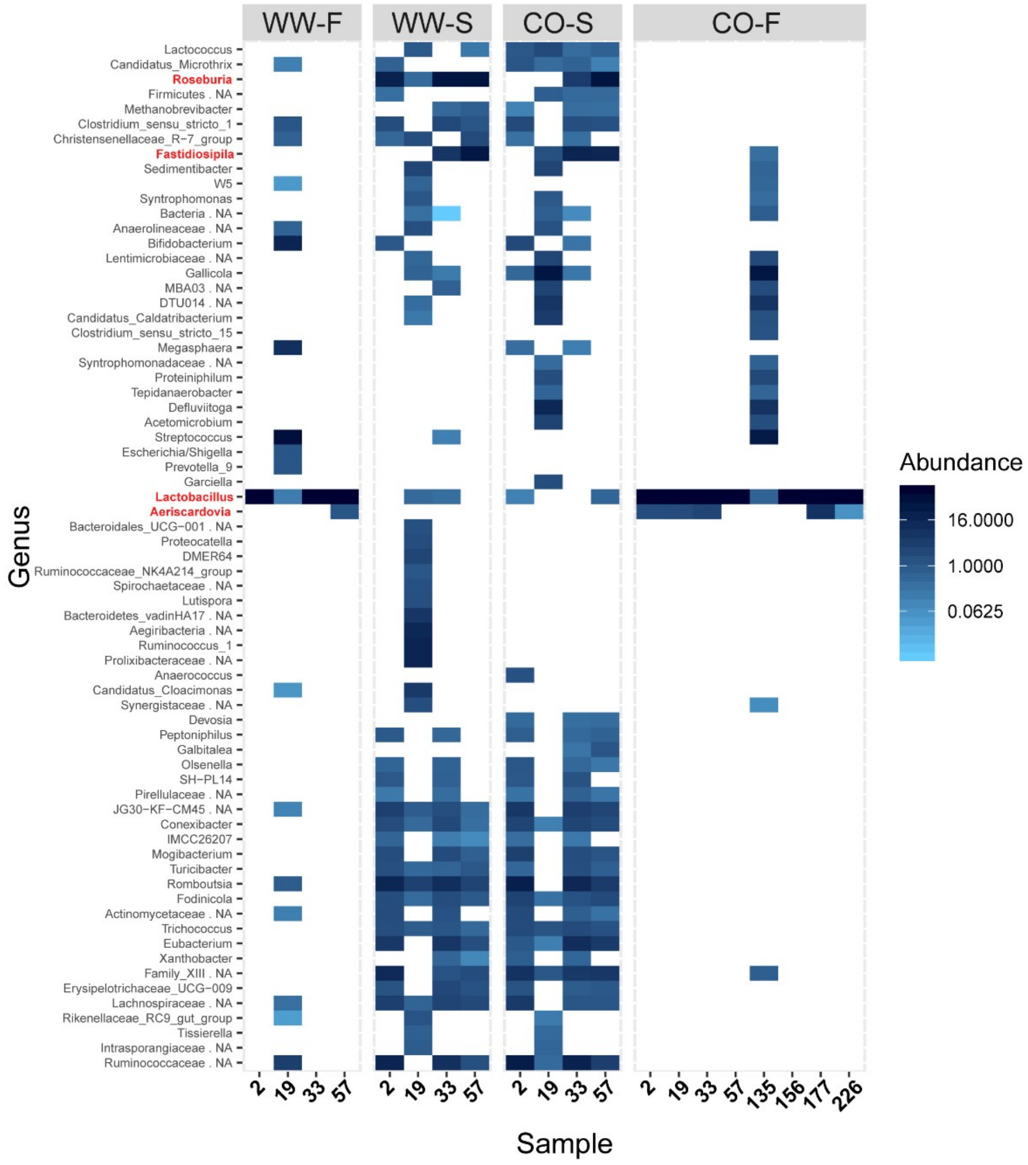

Figure S1. Heatmap illustrating the relative abundance of genera in reactors inoculated with sludge from co-digestion plant (CO) or from an anaerobic digestion process fed mixed sludge (WW), and fed food waste (F) or sewage sludge (S). Genera discussed in the text are shown in red. Days of operation at point of sampling are given on the x-axis.

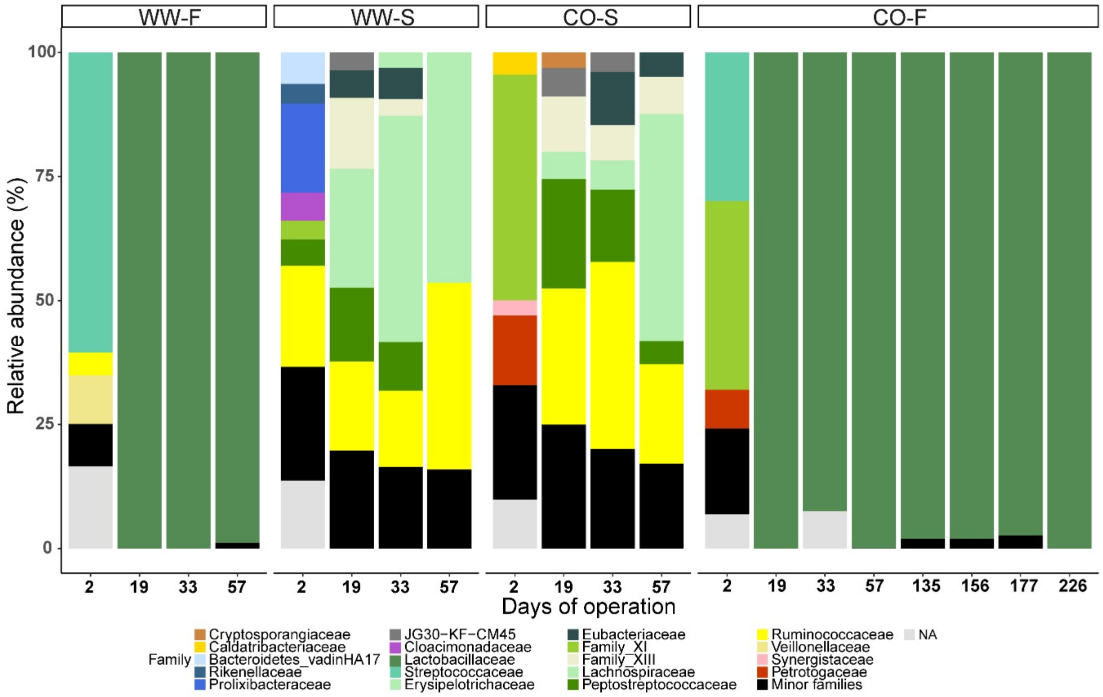

Figure S2. Relative abundance of microbial families (based on total bacterial and archaeal sequences) in reactors inoculated with sludge from co-digestion plant (CO) or from an anaerobic digestion process fed mixed sludge (WW), and fed food waste (F) or sewage sludge (S). Days of operation at point of sampling are given on the x-axis.

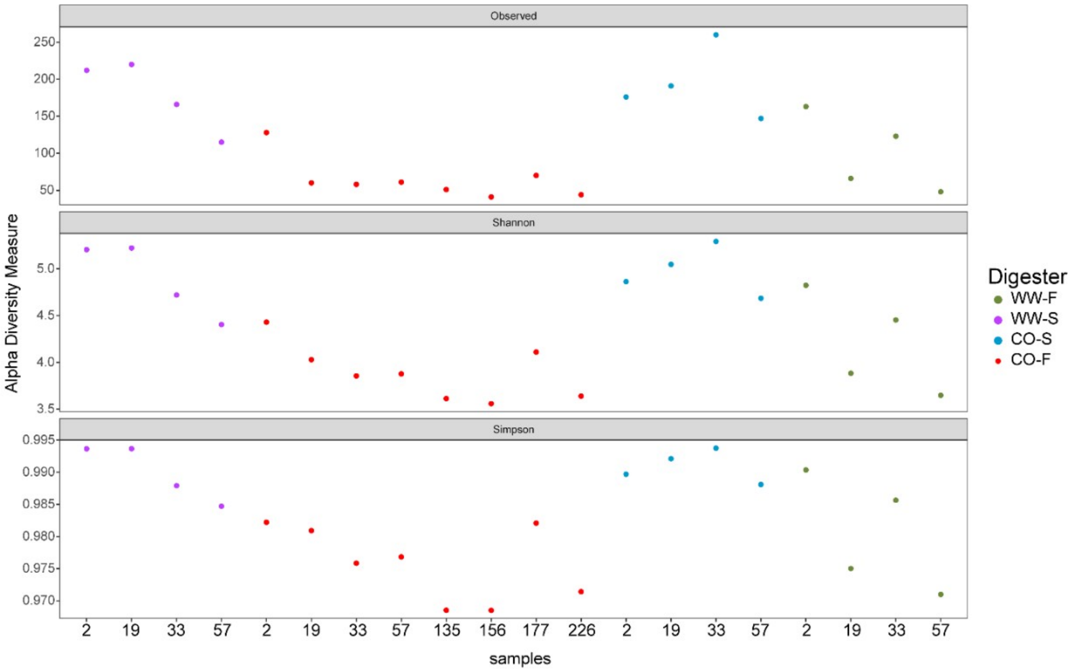

Figure S3. Microbial richness and evenness indices in the digesters inoculated with sludge from co-digestion plant (CO) or from an AD process fed mixed sludge (WW) and fed food waste (F) or sewage sludge (S). Day number in the study period is given on the x-axis.
